# RNA therapeutic targeting of recalcitrant and rare cancers

**DOI:** 10.37349/etat.2026.1002373

**Published:** 2026-05-21

**Authors:** Afeez Adekunle Ishola, Nalini Devi Verusingam, Bashir Lawal

**Affiliations:** IRCCS Istituto Romagnolo per lo Studio dei Tumori (IRST) “Dino Amadori”, Italy; ^1^Developmental Therapeutics Branch (DTB), National Cancer Institute (NCI), National Institute of Health (NIH), Bethesda, MD 20892, USA; ^2^Department of Research and Development, National Cancer Council (MAKNA), Kuala Lumpur 50450, Malaysia; ^3^UPMC Hillman Cancer Center, University of Pittsburgh, Pittsburgh, PA 15260, USA; ^4^Department of Pathology, University of Pittsburgh, Pittsburgh, PA 15260, USA

**Keywords:** recalcitrant cancers, rare cancers, RNA therapeutics, non-coding RNA, DNA drugs

## Abstract

In 2013, more than a decade ago, the “Recalcitrant Cancer Research Act of 2012” was signed into law in the USA. Recalcitrant cancers are among the leading causes of global cancer morbidity and mortality. At the inception of the act, priority was placed on lungs and pancreatic cancers. Despite the tremendous advancement achieved in the research and treatment of said ‘recalcitrant cancers’ in the form of developing novel or modified small molecule and antibody drugs, modest improvement has been recorded for patients’ survival. Also, current mortality and morbidity for recalcitrant cancers keep increasing. Similarly, rare cancers only enjoy very meager research and drug development efforts globally. Consequently, very limited advancement has been made towards therapeutic development targeting rare cancers. Hence, the current situation calls for re-strategizing research efforts and exploring different treatment modalities towards combating recalcitrant and rare cancers. On this note, RNA therapeutics strategy holds a unique and vital prospect because of its propensity to target coding and non-coding RNA transcripts in the biological system. Moreover, RNA therapeutics such as lncRNAs and circRNAs have been established to even modulate protein expressions and biological phenotypic activity through RNA-protein interactions. Therefore, the current review aimed at summarizing existing literature, clinical trials, and elucidating the important prospect of RNA therapeutics in mitigating the recalcitrant and rare cancers menace.

## Introduction

The Recalcitrant Cancer Research Act of 2012 defined recalcitrant tumors as any cancer type with a 5-year survival of less than 20 percent and kills more than 30,000 people yearly in the USA [1, 2]. On the same note, a cancer is classified as rare when its incidence rate is less than 6 in 100,000 people yearly [3]. At the inception of the Recalcitrant Cancer Research Act, research attention was focused on small-cell lung cancer (SCLC) and pancreatic ductal adenocarcinoma (PDAC) [1]. More than a decade later, advanced SCLC 5-year survival is still 7%, while pancreatic carcinoma is 13% [4]. More precisely, in 2025, total pancreatic cancer incidence, which PDAC is responsible for approximately 90%, was estimated at 67,440 new cases in the USA, while 51,980 deaths were recorded as well [4]. Likewise, the USA lung cancer (including SCLC) predicted incidence in 2024 is 234,580, and 125,070 deaths will be recorded by the end of 2024 [5]. Obviously, oncology scientists have not relented in their effort of researching into the molecular events driving the pathogenesis of SCLC and PDAC. Neither have they boycotted researching into therapeutic strategies necessary to curb SCLC and PDAC as a debilitating disease. For instance, a plethora of articles have been published since 2013 to date. Likewise, hundreds of clinical trials have been initiated and/or completed during the same period.

To combat SCLC, National Cancer Institute scientists led a group that reported screening sixty-three human SCLC lines’ response to 103 USA Food and Drug Administration (FDA)-approved oncology agents and 423 investigational agents [6]. The genomic, epigenomic, and miRNA data generated were deposited in a database shared with the SCLC research community [6]. Notwithstanding, these research efforts have translated to modest therapeutic advancement in the form of patients’ survival [7]. Of note, Gazdar and Minna [2] succinctly summarized important research questions needed to be answered to achieve sustainable long-term SCLC treatment, which will translate into better patient survival:

- What is the detailed action mechanism of the initial chemosensitivity and/or subsequent chemoresistance of SCLC [2]?
- Could chemo-resistant SCLC tumors be resensitized and made sensitive?
- How does SCLC escape immune responses, and how to reinstate antitumor immunity [2]?

More recently, Sen [8] attributed recalcitrant cancers’ unsuccessful advancement in treatment to “lack of knowledge regarding our understanding of actionable targets in SCLC and the rapid development of drug resistance by this cancer”. Likewise, the 2019 reports of Clinical Trials and Translational Research Advisory Committee (CTAC)’s SCLC and PDAC progress working groups also corroborated this resolution [9, 10]. In these reports, SCLC and PDAC progress working groups identified research gaps, including new diagnostic approaches, mechanistic pathogenesis, and/or resistance investigation, as well as new therapies development [9, 10]. Altogether, this shows that there are still lapses in our understanding of the molecular pathogenesis of SCLC and PDAC. Unraveling detail molecular pathogenesis will foster better application of currently available small molecules and protein drugs. Simultaneously, opening avenue for developing more modalities targeting the molecular vulnerability of SCLC and PDAC.

Clearly, an additional therapeutic tool is needed to combat recalcitrant and rare cancers. RNA-based therapeutics present a powerful prospect in this regard. A cogent capability of RNA-based therapies is their ability to target coding and non-coding transcripts in biological systems, which broadens cancer druggable targets [11]. Moreover, some RNA transcripts, such as circular RNAs (circRNAs) have longer half-life than small molecules and protein drugs; hence, longer therapeutic residence time in the biological system [12]. Interestingly, therapeutic RNA modalities have been discovered to modulate several stages of cancer hallmarks and even the microenvironment [12–14]. Clinically, Wang et al. [15] recently reviewed in detail various FDA-approved RNA therapeutics, including those under development for cancers and other disease treatments. In this review, we will attempt to summarily identify research lapses contributing to lagging advancement in recalcitrant and rare cancers’ therapeutic outcomes. Likewise, elaborate on the enormous prospect RNA therapies could offer towards achieving the aim of the “Recalcitrant Cancer Research Act of 2012”.

## Comparative statistics and treatment advancement from 2010 to 2025 in USA

### Recalcitrant cancer: SCLC

**Figure 1:**
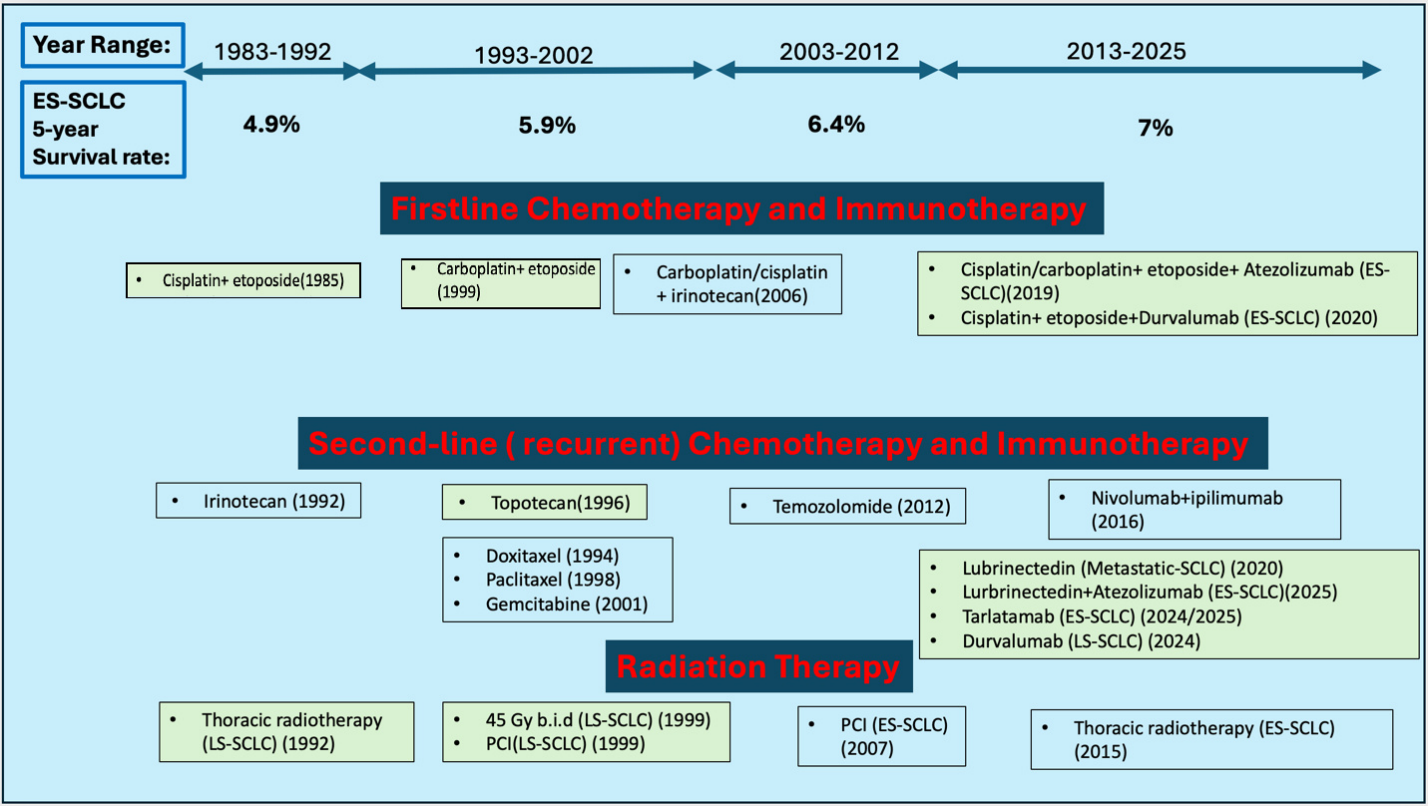
**Schematic timeline of SCLC FDA-approved therapies and 5-year patients’ survival in the last four decades [17, 19].** National Comprehensive Cancer Network (NCCN) recommended therapies are presented in unshaded boxes, while full FDA-approved therapies are shaded in green boxes [17]. ES-SCLC: extensive-stage small-cell lung cancer; LS-SCLC: limited-stage small-cell lung cancer; PCI: prophylactic cranial irradiation.

Lung cancer remains the most prevalent new cancer incidence globally, and SCLC is responsible for about 13% of those new cases [4]. SCLC is highly aggressive, estimated to kill an estimated 250,000 people worldwide every year [16]. SCLC is basically classified into two main groups, namely limited-stage SCLC (LS-SCLC) and extensive-stage SCLC (ES-SCLC). Based on this classification, SCLC treatment differs. For LS-SCLC, standard therapy includes cisplatin/etoposide chemotherapy combined with radiotherapy [17]. A prophylactic cranial irradiation (PCI) is also administered to prevent future metastasis to the brain [17]. Mainly for ES-SCLC, current frontline treatment includes the administration of chemotherapeutic drugs cisplatin and etoposide in combination with immunotherapeutic drugs targeting immune checkpoint inhibitors (programmed death-ligand 1[PD-L1] or cytotoxic T-lymphocyte-associated protein 4 [CTLA-4]) [17]. A comparative timeline of the treatment advancement and patients’ survival is depicted in Figure 1. Likewise, Tariq et al. published an updated management of SCLC [18].

Consequently, although the overall incidence and mortality rate of SCLC are gradually decreasing in the USA, a commensurate increase in SCLC patients’ 5-year survival remains approximately 7%, a little improvement from 6.4% reported between 2002 and 2012 [19, 20]. Of note, recent advances in the understanding of SCLC tumor biology and/or immunity led to the development of immunotherapies with/without chemotherapy. This, combined with better detection techniques and awareness campaigns, might play a part in the modest improvement in ES-SCLC 5-year patient survival stated above. Scientists and other oncology health practitioners rightfully identified the imminent need to develop new therapeutic modalities, such as RNA therapeutics discussed below.

### Recalcitrant cancer: PDAC

Globally, PDAC accounts for 90% of total pancreatic cancer and is currently the 4th leading cause of cancer-related deaths [21], while in the USA, it has surpassed breast cancer to become the 3rd leading cause of cancer-related mortality [22]. The treatment strategy for PDAC patients, like most aggressive carcinomas, depends on disease stage or advancement at detection. Treatment options invariably determine the ultimate outcomes in the form of patient survival. Unfortunately, 90 percent of PDAC cases are detected at an advanced disease stage characterized by high disease aggression, poor prognosis, and low survival [21, 22]. In general, PDAC treatment consists of a combination of surgical resection and multi-agent systemic chemotherapy [23]. First-line treatment involves the administration of 5-fluorouracil (5-FU), leucovorin, irinotecan, and oxaliplatin (FOLFIRINOX) [22, 23]. Meanwhile, other chemotherapies such as Gemcitabine or a combination of gemcitabine and paclitaxel are administered as a second-line treatment [22, 23]. Only a small fraction of PDAC patients (15–20%) qualify for and benefit from surgical resection [22, 23].

**Figure 2:**
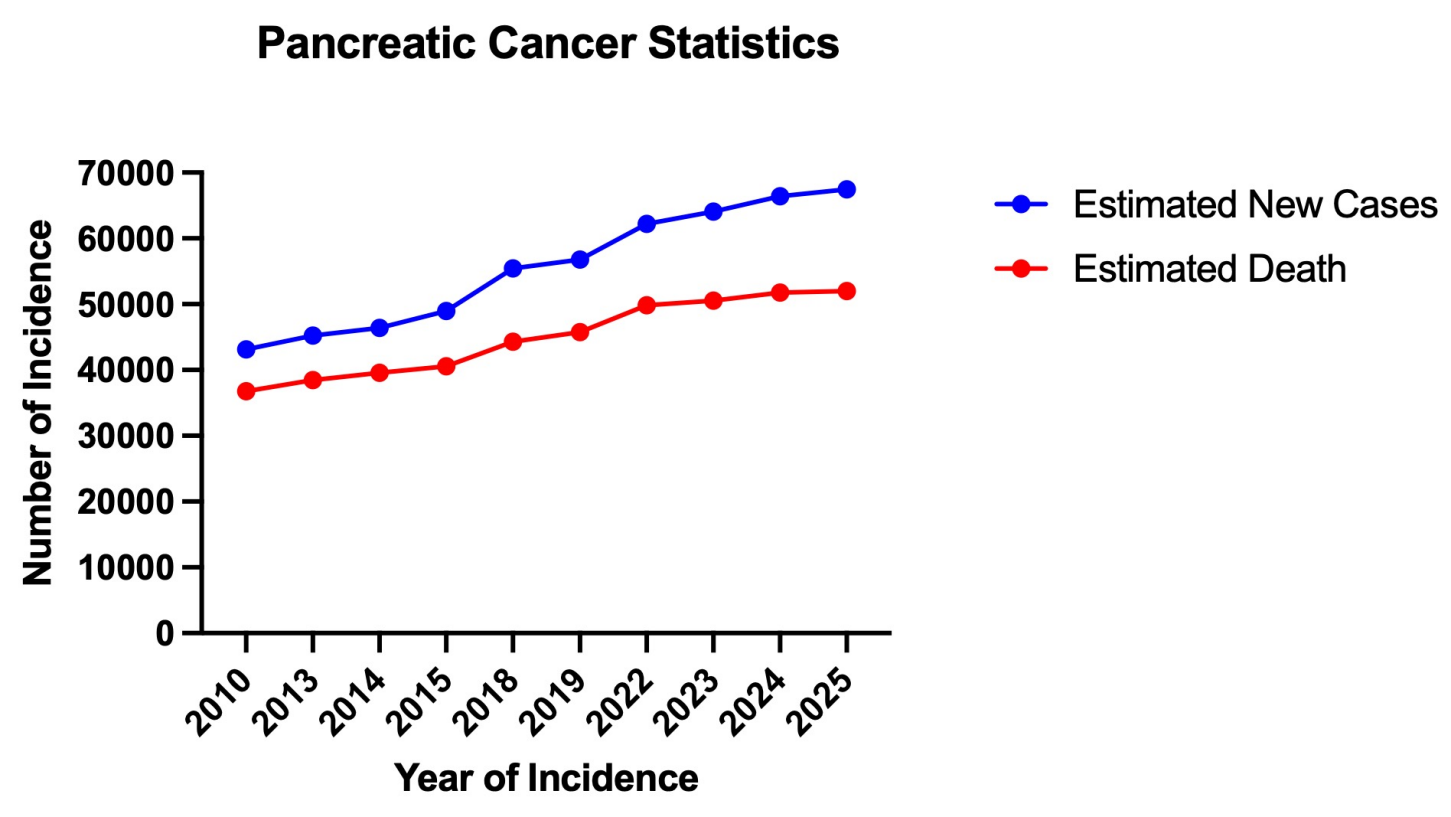
Trends of pancreatic cancer, including Pancreatic Ductal Adenocarcinoma (PDAC), estimated new cases and deaths in the USA within the last 15 years [5, 28–36].

Figure 2 shows the trend of pancreatic cancer, including PDAC new incidence and mortality rate in the last 15 years, while Table 1 summarizes gender specific statistics of pancreatic cancer, which PDAC accounts for approximately 90%, in the USA within the same period. Considering all stages of PDAC, in the USA, overall PDAC 5-year survival still stands at 13% [4, 21–24]. Meanwhile, when PDAC is detected early (stage IA) through adequate surveillance of patients with familial gene susceptibility or abdominal imaging for other ailments, the 5-year survival of patients increases to 20% or higher [25]. Looking back 15-years, all stages PDAC 5-year survival in the year 2010 was 6% [26] compared to 13% in the year 2025 [4] as reported by the American Cancer Society. That is literally a 116% increase in patients’ survival. Despite this remarkable improvement, scientists have identified more strategies that will promote improvement and drive further increase in PDAC patients’ survival, such as intensifying research efforts to understand PDAC at the molecular level with a focus on novel treatment agents preventing the growth, metastasis, and invasion of the disease [21, 27]. Likewise, novel tumor-targeted therapies, including new-generation immunotherapies and RNA therapies, might help [23].

**Table 1:** Gender specific statistics of new cases and mortality of pancreatic cancer, including Pancreatic Ductal Adenocarcinoma (PDAC), in the USA within the last 15 years [5, 28–36].

**Year**	**Estimated new cases**			**Estimated death**		
	**Total new cases**	**Male**	**Female**	**Total death**	**Male**	**Female**
2010	43,140	21,370	21,770	36,800	18,770	18,030
2013	45,220	22,740	22,480	38,460	19,480	18,980
2014	46,420	23,530	22,890	39,590	20,170	19,420
2015	48,960	24,840	24,120	40,560	20,710	19,850
2018	55,440	29,200	26,240	44,330	23,020	21,310
2019	56,770	29,940	26,830	45,750	23,800	21,950
2022	62,210	32,970	29,240	49,830	25,970	23,860
2023	64,050	33,130	30,920	50,550	26,620	23,930
2024	66,440	34,530	31,910	51,750	27,270	24,480
2025	67,440	34,950	32,490	51,980	27,050	24,930

### Clinical development of RNA drugs targeting SCLC and PDAC

The World Health Organization (WHO)’s International Clinical Trials Registry Platform (ICTRP) is a unified public database for information about global clinical trials. The ICTRP data is contributed by 20 clinical trials registries around the world. Using a combination of keywords such as “SCLC and RNA drug” and “PDAC and RNA drug” returned no results on the ICTRP search portal. Meanwhile, search for “RNA drug” on the ICTRP portal returned 120 clinical trial results with none specifically investigating RNA-based modality for SCLC or PDAC. Notwithstanding, a phase 1 clinical trial from China with registration number ChiCTR2200056172 was observing and evaluating the safety of RNA tumor vaccine injection alone or in combination with Programmed cell death protein 1 (*PD-1*) inhibitor for the treatment of Kirsten rat sarcoma viral oncogene homolog (*KRAS*)-mutant advanced solid tumors. Since the majority of PDAC cases are *KRAS* mutation-driven, this trial might consider recruiting PDAC patients. Although this trail was last updated in August, 2024. Similarly, a European phase 1/2a clinical trial (EUCTR2019-004323-20-DE) was evaluating the safety and preliminary efficacy of Claudin 6 (CLDN6) chimeric antigen receptor T-cell therapy (CAR-T) with or without an RNA vaccine (CLDN6 RNA-LPX) in patients with CLDN6-positive relapsed or refractory advanced solid tumors. Although no specific mention of PDAC and SCLC, their inclusion criteria stated that the patient must have a histologically confirmed solid tumor that is metastatic or unresectable. SCLC and PDAC patients might qualify for this clinical trial. This trial was also last updated in July, 2024. ICTRP can be accessed at the web address (https://trialsearch.who.int/Default.aspx). Further pairing of specific RNA forms, such as small interfering RNA (siRNA), long non-coding RNA (lncRNA), circRNA, antisense oligonucleotide (ASO), and microRNAs (miRNAs) with PDAC or SCLC did not return any result.

We further specifically searched Clinicaltrials.gov, the largest clinical trial registry in the world, using the same keywords as explained above: NCT03608631 was discovered. NCT03608631 is a phase I study of mesenchymal stromal cell-derived exosomes with *KRASG12D* siRNA for metastatic pancreatic cancer patients harboring the *KRASG12D* mutation. This trial is still recruiting and is expected to be concluded in April, 2027. On the same note, we investigated pipelines of top RNA therapeutics companies and start-ups such as Moderna, BioNTech, Orna Therapeutics, Anylam Therapeutics, and so on. Our research showed that Moderna has the most advanced clinical trials for developing an oncology-based RNA drug. Unfortunately, SCLC and PDAC are not included in those cancers under Moderna’s investigative drug discovery pipeline. Therefore, so far, clinical development efforts of RNA drugs for recalcitrant cancers such as SCLC and PDAC are very limited. This further substantiates the call for improved exploitation of RNA therapeutic platforms in combating debilitating diseases known as SCLC and PDAC.

Our discussion so far has summarily present literatures about the introduction, classification, statistics, clinically approved treatment modalities, and development of prospective RNA drugs for recalcitrant cancers such as SCLC and PDAC. It is obvious that current research efforts towards discovering long-lasting efficacious treatment for SCLC and PDAC can benefit tremendously from exploring RNA therapeutic modalities. On the same note, rare cancers are known to receive even less research attention. Therefore, we will attempt to provide a brief background on rare cancers and summarise current literature about rare cancer diagnosis, statistics, clinical treatment, and the prospect of RNA drug exploitation in developing more effective treatment for certain rare cancers.

## A preface into rare cancers

Rare cancers are defined as malignancies occurring in fewer than 6 cases per 100,000 individuals annually. These cancers pose unique challenges in diagnosis, treatment, and research funding while collectively accounting for approximately 20% of all cancer diagnoses [36]. These malignancies are often underrepresented in clinical trials due to their rarity and the associated logistical challenges [36]. In the last decade, advancements in molecular biology, genomics, and therapeutic modalities have started to bridge the gap in rare cancer management [3]. For instance, next-generation sequencing (NGS) has enabled the identification of rare oncogenic mutations, facilitating the development of targeted therapies. Additionally, the emergence of immune checkpoint inhibitors has reshaped the treatment landscape for cancers previously considered untreatable [3]. Breakthroughs in RNA-based therapeutics, such as FDA approval of Patisiran for hereditary amyloidosis, highlight the clinical potential of RNA technologies in addressing rare diseases, including rare cancers [37, 38]. RNA therapeutics, including siRNA, ASOs, and mRNA vaccines, have emerged as a promising frontier due to their ability to target undruggable pathways and genes. For instance, siRNA therapies have successfully silenced *KRAS* mutations in preclinical pancreatic cancer models, leading to tumor regression [36]. Similarly, ASOs targeting MYC family protein (MYC) mRNA have demonstrated significant inhibition of tumor growth in aggressive lymphomas [36]. Furthermore, mRNA vaccines encoding neoantigens have shown robust immune activation against melanoma and colorectal cancers in early clinical trials [39].

### Comparative statistics over two decades

A long-term analysis of rare cancer trends highlights both challenges and progress in incidence and survival outcomes. Over the past two decades, advancements in diagnostic techniques, including molecular imaging and NGS, have contributed to earlier detection and improved characterization of rare cancers. Simultaneously, therapeutic innovations such as immunotherapies, targeted therapies, and RNA-based treatments have significantly enhanced survival rates across various cancer types. The incidence and survival trends for rare cancers (Table 2) underscore the gradual improvements in survival outcomes for rare cancers over two decades, driven by advancements in early detection, targeted therapies, and supportive care innovations. However, the modest increases in incidence highlight the ongoing need for research into prevention and environmental or genetic risk factors. The following table, derived from the Global Cancer Observatory and SEER Database, provides a comprehensive comparison of incidence rates and 5-year survival rates from 2003, 2013, and 2023, illustrating these trends [36, 40].

**Table 2:** Incidence and survival trends for rare cancers (2003–2023).

**Cancer type**	**Incidence (per 100,000) 2003**	**Incidence (per 100,000) 2013**	**Incidence (per 100,000) 2023**	**Survival rate (5-year) 2003**	**Survival rate (5-year) 2013**	**Survival rate (5-year) 2023**
Brain cancers	4.1	4.3	4.5	30%	35%	42%
Bone cancers	0.8	0.9	1.0	50%	55%	63%
Pediatric cancers	14.0	15.2	16.0	65%	70%	80%
Leukemia	12.5	13.7	14.5	55%	60%	75%

### Clinical trial improvement in the last decade

Clinical trials for rare cancers have historically faced challenges, including small patient populations, heterogeneous disease presentations, and limited funding. However, recent innovations have addressed these barriers, leading to significant progress in drug development and patient outcomes. The data presented in this section are derived from analyses of publicly available clinical trial databases, including ClinicalTrials.gov and the WHO’s ICTRP, as well as peer-reviewed publications and industry reports [41–43].

### Advances in rare cancer research

**Adaptive Trial designs:** Clinical trial platforms such as the National Cancer Institute’s Molecular Analysis for Therapy Choice (NCI-MATCH) and the Targeted Agent and Profiling Utilization Registry (TAPUR) allow for the inclusion of rare cancer subtypes by utilizing shared control groups and flexible endpoints. These designs improve efficiency and reduce the burden on small patient populations. A pivotal study by [44] demonstrated that NCI-MATCH successfully matched patients with rare cancer subtypes to targeted therapies based on genetic profiles, leading to improved treatment outcomes. Additionally, the TAPUR trial has shown the efficacy of repurposing FDA-approved drugs for off-label use in rare cancers with specific molecular targets [45, 46].

**Biomarker-driven studies:** The identification and validation of molecular biomarkers, including RNA-based signatures, have enabled patient stratification, maximizing trial success rates by targeting responsive subgroups. For example, the use of *PD-L1* expression as a predictive biomarker in immune checkpoint inhibitor trials has transformed treatment approaches for cancers like Merkel cell carcinoma, a rare skin cancer [47, 48].

**Global collaboration:** International consortia such as Expert Care for Rare Adult Solid Cancer (EURACAN) and Rare Cancers Europe have pooled resources to conduct larger, more robust trials, thereby addressing challenges associated with limited patient enrollment. This collaborative approach has also improved data sharing and standardization of protocols. A notable success was the EURACAN-led study on sarcomas, which pooled over 500 patients across 20 countries, resulting in the development of standardized treatment guidelines and identification of novel therapeutic targets [49, 50]. Moreover, Rare Cancers Europe has advanced policy changes to increase funding and collaboration opportunities for rare cancer research.

These key advances underscore the importance of leveraging innovative trial designs, biomarkers, and global networks to overcome the inherent challenges of rare cancer research. As the field of RNA therapeutics continues to evolve, integrating these strategies into trial frameworks will be critical for accelerating the development of effective therapies and improving patient outcomes.

### Advances in RNA-based therapeutics for rare tumors

#### Brain cancers

Brain cancers, including glioblastoma (GBM) and anaplastic astrocytomas, remain among the most challenging malignancies due to their aggressive nature, heterogeneity, and resistance to conventional therapies like surgery, radiation, and chemotherapy. Recent advances in RNA-based therapeutics have introduced novel approaches that specifically target molecular drivers and tumor microenvironments:

1. **siRNA and miRNA therapies:** siRNAs and miRNAs are being employed to silence key oncogenes and regulate pathways critical to tumor growth. For example, epidermal growth factor receptor variant III (EGFRvIII), a common mutation in GBM, has been targeted using siRNAs, leading to reduced tumor proliferation in preclinical models. Similarly, miRNA mimics or inhibitors (e.g., miR-21 inhibition) have shown promise in restoring tumor suppressor pathways [51, 52]. Early-phase clinical trials are exploring these approaches, but delivery remains a major hurdle. A 2024 study investigated siRNAs targeting metabolic pathways such as glutaminase in GBM, resulting in reduced tumor growth in preclinical models. These findings suggest metabolic reprogramming as a viable target for RNA therapeutics [53].
2. **mRNA vaccines combined with checkpoint inhibitors:** Personalized mRNA vaccines targeting tumor-associated antigens (TAAs) or neoantigens are under development. In a pivotal trial, mRNA vaccines encoding patient-specific neoantigens demonstrated robust T-cell activation, resulting in prolonged progression-free survival in a subset of GBM patients [54]. These vaccines are often combined with immune checkpoint inhibitors to enhance efficacy. A recent study demonstrated significant survival benefits when personalized mRNA vaccines were combined with PD-1 inhibitors in patients with recurrent GBM [55]. This combination therapy enhanced T-cell infiltration and reduced tumor burden compared to mRNA vaccines alone.
3. **ASOs:** Therapies such as nusinersen, originally developed for spinal muscular atrophy (SMA), are being repurposed for brain tumors. ASOs targeting oncogenic splicing variants, such as TERT promoter mutations, have shown potential to inhibit tumor progression. New-generation ASOs with enhanced blood-brain barrier penetration are currently under investigation.
4. **Clustered regularly interspaced short palindromic repeats**-**associated protein (CRISPR-Cas) gene editing:** CRISPR-Cas systems guided by RNA have demonstrated the ability to knock out key oncogenic drivers like phosphatase and tensin homolog (PTEN) deletions and *IDH* mutations in GBM [56, 57]. Preclinical studies have highlighted significant tumor regression using CRISPR-Cas approaches delivered via nanoparticle carriers [58]. A novel lipid nanoparticle (LNP) system for delivering CRISPR components was reported in 2023 to improve editing efficiency in GBM xenograft models. This approach specifically targeted *O^6^-methylguanine-DNA methyltransferase* (*MGMT*) promoter methylation, sensitizing tumors to alkylating agents [59].
5. **Exosome-based RNA delivery:** Exosomes, natural extracellular vesicles, are being engineered to deliver RNA therapeutics specifically to brain tumors. These systems show improved delivery efficiency and reduced off-target effects, overcoming challenges of systemic toxicity often seen with traditional methods. Clinical trials initiated in 2024 are testing exosome-mediated delivery of siRNAs targeting oncogenes such as *Bcl-2*. Preliminary results show reduced systemic toxicity and enhanced tumor-specific uptake [60, 61].
6. **Clinical outcomes and challenges**: Despite significant advancements, the clinical translation of RNA-based therapies for brain cancers faces challenges. Limited penetration of the blood-brain barrier, heterogeneity in tumor microenvironments, and immunosuppressive niches within the brain reduce therapeutic efficacy. Nevertheless, the integration of RNA therapeutics with conventional treatments, such as radiation and immunotherapy, shows promise in overcoming these obstacles. Recent clinical trials, such as those exploring siRNA targeting *Bcl-2* or mRNA vaccines encoding neoantigens, have reported partial responses and improved overall survival in early-phase studies. However, more extensive, randomized trials are needed to validate these findings and address delivery challenges.

#### Bone cancers

Bone cancers, such as osteosarcoma and Ewing sarcoma, disproportionately affect younger populations and often exhibit resistance to standard chemotherapies. RNA therapeutics have provided new avenues:

1. **siRNA-based approaches:** Targeting genes like mouse double minute 2 (MDM2) and vascular endothelial growth factor (VEGF) using siRNAs has demonstrated efficacy in preclinical models by reducing tumor growth and angiogenesis [62]. Studies from 2023 explored siRNAs delivered via LNPs to improve specificity and reduce systemic toxicity [63].
2. **Exosome-mediated delivery:** Exosomes derived from mesenchymal stem cells (MSCs) are being developed to deliver RNA therapeutics to bone tumors. Present studies also demonstrated enhanced delivery of siRNAs targeting anaplastic lymphoma kinase (*ALK*) fusion genes in Ewing sarcoma, leading to significant tumor regression in mouse models [64].
3. **mRNA vaccines:** Personalized neoantigen-based mRNA vaccine approaches are being explored within pediatric sarcoma consortia, though no osteosarcoma-specific mRNA vaccine has yet entered a late-phase trial [64].
4. **CRISPR gene editing:** CRISPR-based approaches targeting *EWS-FLI1* fusion genes in Ewing sarcoma have shown success in preclinical studies, with novel delivery methods under investigation for translation into clinical trials [64].
5. **Clinical outcomes and challenges:** Despite these advances, challenges such as delivery to bone tissue and immune evasion by tumors remain. Current clinical trials are exploring combinations of RNA-based therapies with conventional chemotherapies to enhance efficacy and reduce relapse rates.

#### Pediatric cancers

Pediatric cancers, while rare, remain the leading cause of disease-related death in children. RNA-based therapies are revolutionizing treatment paradigms:

1. **Fusion oncoprotein targeting:** ASOs targeting fusion genes (e.g., paired box gene 3-forkhead box O1 [*PAX3-FOXO1*] in rhabdomyosarcoma) are under development. Recent findings from 2023 demonstrated inhibition of tumor growth in preclinical models.
2. **mRNA vaccines for relapse prevention:** Trials are exploring mRNA vaccines for preventing relapse in high-risk neuroblastoma and medulloblastoma patients. Early-phase studies have shown promising immune activation and tolerability profiles [65].
3. **Epigenetic modulation:** RNA-targeting compounds are being used to restore normal expression of tumor suppressor genes silenced by methylation [66].

#### Leukemia

Leukemias, characterized by uncontrolled proliferation of hematopoietic cells, have seen significant advancements with RNA therapeutics:

1. **ASOs and splice modulation:** Therapies like olaptesed pegol inhibit C-X-C motif chemokine receptor 4 (*CXCR4*) signaling, reducing leukemia stem cell survival [67]. Studies in 2022 expanded ASO applications to target *Bcl-2* and MYC pathways in acute lymphoblastic leukemia (ALL).
2. **CAR-T enhancements:** RNA engineering is being used to improve chimeric antigen receptor (CAR) T-cell therapies, increasing their efficacy and reducing off-target effects. Recent trials in 2024 demonstrated enhanced CAR-T durability through RNA modifications [68].
3. **RNA vaccines:** Investigational RNA vaccines targeting neoantigens in acute myeloid leukemia (AML) showed robust T-cell responses and prolonged survival in mouse models [69].

RNA therapeutics represent a transformative approach to targeting recalcitrant and rare cancers. Their ability to modulate gene expression, target specific molecular pathways, and induce immune responses positions them as key players in the fight against these challenging malignancies. Continued advancements in delivery systems, clinical trial design, and biomarker discovery will be critical to unlocking their full potential. Hence, subsequent sections will elaborate more on the detailed historical foundation and translational breakthrough of RNA therapeutics in general. To further establish the enormous potential of RNA drugs development for recalcitrant and rare cancers, dedicated section will present FDA-approved clinical success stories of RNA therapeutics in non-malignant diseases. More interestingly, we will reiterate few ongoing research efforts towards developing oncology-related RNA drugs and in extension recalcitrant and rare cancers.

### The emergence of RNA therapeutics: historical foundations and translational breakthroughs

Ground breaking discovery in RNA biology gained its momentum in 1961 when Jacob and Monod [70] theorized the presence of mRNA. By the 1990s, miRNAs were recognized for their role in post-transcriptional gene silencing [71], followed by the discovery of small nucleolar RNAs (snoRNAs), piwi-interacting RNAs (piRNAs) [72], lncRNAs [73–76], and recently circRNAs [77, 78]. These breakthroughs have certainly redefined the roles of RNA in biology, offering novel perspectives on genetic regulation and diseases.

Building on previous cutting-edge research, RNA-based therapeutics have progressively advanced the landscape of medicine, resolving unmet clinical needs through precise and innovative technologies. Key RNA therapeutic classes discovered over the years include ASOs, RNA aptamers, siRNAs, mRNA vaccines, and lipid-based nanotechnology. ASO technology was first introduced in 1978 when Zamecnik et al. [79] and Stephenson et al. [80] demonstrated its potential to suppress *Rous sarcoma* virus activity in chicken embryos. After two decades of extensive research in ASO, these efforts led to the FDA approval of Fomivirsen [81], the first RNA-based therapeutics delivered via intravitreal injection, for the treatment of cytomegalovirus (CMV) retinitis in AIDS patients [81].

After the approval of ASO, RNA research and therapeutic efforts intensified towards combating mostly non-oncology diseases. Another notable historical discovery includes RNA aptamer-based drug, Macugen (pegaptanib), for treating age-related macular degeneration (AMD), marking a significant milestone in its clinical application [82]. In 2004, the first siRNA clinical trial, AGN211745 (siRNA-027), was initiated, targeting the VEGF signaling pathway to treat wet neovascular AMD [83]. This provided key evidence for the therapeutic potential of RNA interference (RNAi) using siRNAs as therapeutic tools. Subsequently, the FDA approved the first siRNA-based drug, Patisiran, in 2018 [84]. CRISPR technology also ushered in a powerful therapeutic system, which combined the enzymatic activity of an endonuclease with single guide RNA (sgRNA) to target disease-associated genetic (DNA and RNA) targets in various prokaryotic and eukaryotic biological systems. In 2023, the FDA approved exagamglogene autotemcel (exa-cel), the first CRISPR-based therapy, marking a major milestone in genetic medicine [85].

The most practical application of RNA therapeutic importance was witnessed during the COVID-19 pandemic in 2020. The principle of mRNA vaccines is based on the use of messenger RNA to instruct cells to produce a viral protein, thereby stimulating an immune response. mRNA vaccines deliver the genetic blueprint for the spike protein of the severe acute respiratory syndrome coronavirus 2 (*SARS-CoV-2*) virus in LNPs, prompting cells to generate the protein and trigger an immune reaction [86]. This approach enables rapid vaccine development and facilitated the swift development of mRNA vaccines during the COVID-19 pandemic, contributing to the 2020 release of the Pfizer-BioNTech and Moderna COVID-19 vaccines [87].

LNP technology, developed in the early 1970s by scientists Gregoriadis and Ryman [88, 89], is a crucial advancement in RNA therapeutics for efficient RNA delivery. LNPs, nanoscale lipid-based carriers, encapsulate RNA molecules like siRNAs, protecting them from degradation and facilitating targeted delivery to specific tissues [90, 91]. This innovative delivery system has revolutionized RNA-based therapies, making them viable for treating various diseases, including genetic disorders and cancers. LNPs enhance the stability, efficiency, and tissue-specific targeting of RNA molecules, providing a foundation for high-impact clinical applications [92]. The clinical success of LNP-formulated RNA therapeutics is demonstrated by several FDA-approved drugs: Patisiran, which was the first LNP-formulated siRNA drug to treat hereditary transthyretin-mediated amyloidosis (hATTR), a rare genetic disorder [84]. Givosiran targets aminolevulinic acid synthase 1 (ALAS1) for acute hepatic porphyria (AHP), another rare metabolic disorder [93]. In 2023, Nedosiran (Rivfloza) was approved, targeting lactate dehydrogenase A (LDHA) for primary hyperoxaluria type 1 (PH1) [94]. These approvals underscore the transformative potential of LNP-based RNA therapeutics in treating genetic and metabolic diseases, highlighting LNP technology as a key enabler in advancing RNA-based treatments.

### Validated RNA therapeutic platforms in non-malignant diseases

RNA-based therapies are an emerging and rapidly advancing field, particularly in the treatment of rare diseases. Although RNA-based approaches show growing promise for targeting difficult-to-treat cancers, their application in oncology remains largely limited to ongoing clinical trials. While the FDA’s approval of certain RNA therapies highlights their potential, their full impact, safety concerns, and long-term effectiveness are yet to be fully established in recalcitrant cancers. Here are some notable examples of successful RNA-based treatments in rare diseases. A full list is presented in Table 3 below.

**Table 3:** Trends in clinical trials for rare cancers.

**Cancer type**	Number of trials (2013)^*^	Number of trials (2023)^*^	**Trial types**	**Median sample size**	**Trial success rate (phase III)**
Brain cancers	45	120	Adaptive, biomarker-driven, immunoRNA	85	28%
Bone cancers	15	50	Targeted, RNA-based, exosome delivery	50	32%
Pediatric cancers	120	250	Fusion targeting, mRNA vaccines	70	45%
Leukemia	200	500	CAR-T enhancements, RNA vaccines	100	55%

^*^The number of clinical trials conducted in 2013 and 2023 for each cancer type was extracted from ClinicalTrials.gov and WHO ICTRP databases using relevant keywords (e.g., “brain cancer,” “bone cancer,” “pediatric cancer,” “leukemia”) and filtered by trial phase and start date. The data were validated using peer-reviewed publications on the trends in rare cancer research [41]. The classification of trial types was informed by recent reviews of oncology trials [42] and included adaptive trials, biomarker-driven designs, RNA-based therapies, and immunotherapies. Median sample sizes were calculated based on published analyses of rare cancer trials and aggregated from trial datasets. Success rates for Phase III trials were determined from historical data published in oncology-specific meta-analyses [43], which reported an average success rate of 3.4% for all oncology trials, with higher rates observed for rare cancers due to biomarker-driven approaches. CAR-T: chimeric antigen receptor T-cell therapy; ICTRP: International Clinical Trials Registry Platform; WHO: World Health Organization.

#### Nusinersen (Spinraza)—SMA

SMA, a genetic disorder caused by mutations in the survival motor neuron 1 (*SMN1)* gene, leads to a reduction in levels of SMN protein, followed by progressive muscle weakness. Nusinersen, developed by Biogen and approved by the FDA in 2016, is an ASO-based drug. It targets the *SMN2* gene, a paralog of *SMN1* that produces insufficient functional SMN protein due to alternative splicing that excludes exon 7. Nusinersen corrects the defects by binding to a specific sequence in the *SMN2* pre-mRNA, enhancing the inclusion of exon 7 during splicing instead. This correction increases the production of full-length, functional SMN protein, which is critical for the survival and function of motor neurons. The drug administered intrathecally targets the central nervous system and has shown efficacy for SMA in improving motor function and slowing disease progression across all age groups [95].

#### Patisiran (Onpattro)—variant ATTR

Patisiran, developed by Alnylam Pharmaceuticals and approved by the FDA in August 2018, is the first RNAi-based therapeutic for hATTR. This rare genetic disorder, affecting more than 10,000 people worldwide, is caused by mutant TTR protein misfolding, leading to amyloid fibril deposition in organs, primarily affecting cardiac and axonal cells [96]. Patisiran, a double-stranded siRNA encapsulated in LNPs, targets liver-produced TTR mRNA, silencing its translation and reducing pathogenic TTR levels. Administered intravenously every three weeks, Patisiran significantly improves neuropathy, cardiac symptoms, and quality of life, marking a breakthrough in treating hATTR-associated polyneuropathy [97].

#### Givosiran (Givlaari)—AHP

AHP is a rare metabolic disorder triggered by the upregulation of the ALAS1 gene, resulting in the toxic accumulation of aminolevulinic acid (ALA) and porphobilinogen (PBG) in the liver. With an estimated global prevalence of 1 in 100,000 individuals, AHP significantly impacts patient health. Givosiran, an FDA-approved RNAi therapeutic (2019), is delivered subcutaneously using *N*-acetylgalactosamine (GalNAc)-conjugated siRNA to target ALAS1. The RNAi drug effectively inhibits ALAS1 expression and lowers ALA and PBG levels, alleviating symptoms and enhancing quality of life. To date, long-term studies confirm its safety and sustained efficacy over four years [98].

#### Lumasiran (Oxlumo)—PH1

PH1 is a rare genetic disorder characterized by excessive oxalate production due to alanine-glyoxylate aminotransferase deficiency, leading to calcium oxalate kidney stones. Lumasiran, a siRNA targeting hydroxyacid oxidase 1 (HAO1) mRNA, inhibits glycolate oxidase synthesis, reducing glyoxylate and oxalate production. This GalNAc-conjugated siRNA is delivered subcutaneously and approved in 2020 in the EU and USA for all age groups. Lumasiran has demonstrated efficacy in clinical trials, significantly reducing urinary and plasma oxalate levels over six months with a favorable safety profile [99].

#### Casimersen (AMONDYS 45)—Duchenne muscular dystrophy (DMD)

DMD is a severe and progressive genetic disorder caused by mutations in exons 45 through 55 of the *DMD* gene, leading to the absence of dystrophin, a critical protein for muscle stability. This results in muscle degeneration, loss of function, and cardiomyopathy, eventually requiring assisted ventilation and causing premature death. Casimersen, developed by Sarepta Therapeutics, is a phosphorodiamidate morpholino oligomer (PMO) ASO engineered for increased resistance to enzymatic degradation, enhancing its stability and therapeutic efficacy. By binding to exon 45 of *DMD* pre-mRNA, Casimersen facilitates exon skipping during mRNA splicing, enabling the production of a truncated but functional dystrophin protein. Administered via intravenous infusion, Casimersen received FDA approval in 2021, offering a targeted approach to address the underlying rare genetic defect [100].

#### COMIRNATY—Pfizer COVID-19 vaccine

Comirnaty, developed by Pfizer-BioNTech, is a vaccine designed to prevent COVID-19. It received full approval from the U.S. FDA in August 2021 for individuals aged 16 and older. The vaccine utilizes tozinameran, an mRNA molecule, to instruct cells to produce a protein from the original *SARS-CoV-2* strain, stimulating an immune response. To address evolving variants, Comirnaty has been updated to include formulations targeting newer strains, such as Omicron subvariants BA.4/5, XBB.1.5, JN.1, and KP.2. These adapted versions employ specific mRNA molecules, such as Raxtozinameran and Famtozinameran tailored to the respective variants. Encapsulated in LNPs, the mRNA enters host cells, where it directs the synthesis of a modified *SARS-CoV-2* spike protein. This process triggers the immune system to produce neutralizing antibodies and cellular responses, enhancing protection against infection. In phase III trials, Comirnaty demonstrated 95% efficacy against symptomatic COVID-19. Its advancements ensure robust immunity as the virus continues to evolve [101].

#### Spikevax (Moderna COVID-19 vaccine)

On December 18, 2020, the FDA granted emergency use authorization (EUA) for the Moderna COVID-19 vaccine for adults aged 18 and older, making it the second vaccine approved for COVID-19 prevention. The vaccine, later fully approved by the FDA in January 2022, contains synthetic, nucleoside-modified mRNA encapsulated in LNPs. This mRNA encodes the pre-fusion stabilized spike protein of *SARS-CoV-2*, which is critical for viral entry into host cells. Upon vaccination, the spike protein triggers an immune response that protects against the virus. As *SARS-CoV-2* evolved, the vaccine was adapted to target newer variants. Moderna’s Spikevax has since been authorized in several versions, including bivalent vaccines for the Omicron BA.1, BA.4-5, XBB.1.5, and JN.1 subvariants. These adaptations help maintain effective protection against COVID-19 by targeting the most recent strains of the virus, including the JN.1 variant [101].

### Why RNA therapeutics are still limited in rare cancers

RNA-based drugs (siRNA, miRNA, lncRNA, and mRNA vaccines) have made major strides, but most advances and trials are concentrated in common solid tumors (melanoma, NSCLC, prostate, pancreatic) and hematologic malignancies, not rare cancers [102–105]. Several factors explain this gap:

1. **Biology and target discovery:** Many rare cancers lack deep multi‑omic characterization, so the disease‑specific RNA targets (oncogenic miRNAs/lncRNAs, neoantigens for mRNA vaccines) are less well defined than in common tumors. lncRNA targeting in particular depends on cell‑type restricted expression maps that are incomplete for rare entities [106–108].
2. **Delivery and pharmacology challenges:** Efficient, safe delivery of RNA into tumor tissue is still a major bottleneck. Current LNP and other formulations work best for liver and some well‑vascularized tumors, but struggle with deep, heterogeneous solid tumors, which is especially problematic when only very small, dispersed patient populations are available to optimize dosing and delivery. Suboptimal delivery and off‑target effects have already caused termination of several oncology RNA trials [108–113].
3. **Safety, off‑target effects, and incomplete RNA biology:** miRNA and lncRNA therapeutics can act on many transcripts at once, making specificity and toxicity difficult to control, which may lead to early‑phase failures and a cautious regulatory stance. For many ncRNAs (ncRNAs), functional roles in specific rare tumor contexts remain poorly understood, limiting rational drug design [114].
4. **Small, fragmented patient populations and trial design:** Rare cancers make it hard to run adequately powered, stratified RNA trials and to embed complex pharmacodynamic, immune, and biomarker endpoints that RNA agents require [111]. Reviews of ncRNA clinical trials emphasize that, even in common cancers, most work is still early phase with heterogeneous designs; scaling that to many rare indications is a major logistical and financial barrier [104, 106].
5. **Economic and regulatory constraints:** Manufacturing scale‑up, cold‑chain logistics, and evolving regulatory pathways for novel modalities (siRNA, circRNA, engineered lncRNAs) add cost and uncertainty, which companies preferentially absorb in large indications rather than rare tumors. Approved RNA drugs have largely targeted rare genetic diseases where systemic delivery and clear single‑gene targets are more tractable than in heterogeneous rare cancers 618. Overall, the field is still solving platform‑level issues, such as delivery, specificity, safety, and manufacturing, which are being addressed first in more common cancers before being extended to small, rare‑cancer populations.

### Oncogenic RNA therapeutic prospects in recalcitrant and rare cancers now and in the future

Recent progress has positioned RNA therapeutics at the core of a new precision oncology landscape. While earlier RNA medicines primarily targeted inherited or metabolic disorders, current developments are moving into solid and hematologic malignancies, a group of diseases characterized by high fatality and therapeutic recalcitrance. Following the policy impetus of the *Recalcitrant Cancer Research Act of 2012*, multiple RNA oncology programs have entered the clinic [1, 2]. RNA‑based therapeutics indeed represent a promising strategy for treating rare cancers, particularly those that are resistant to conventional modalities such as chemotherapy, radiotherapy, monoclonal antibodies, and immunotherapy. By modulating RNA transcripts, these therapies enable the regulation of oncogene expression, the restoration of tumor suppressor gene function, and the correction of genetic mutations, offering personalized treatment options tailored to the specific genetic alterations driving individual cancers [111]. Furthermore, their non-integrative and degradable nature ensures safety, positioning RNA therapeutics as an innovative alternative to traditional and DNA-based treatments in cancer care [111]. Current research has shown that RNA-based strategies, such as RNAi, RNA-based CAR-T cell therapies, and mRNA vaccines, are being explored for their ability to target key pathways involved in cancer progression. Early-phase clinical trials have demonstrated promising results, particularly in hematologic and solid tumors [115].

To date, over 120 RNA-based cancer trials are active or may have been completed according to ClinicalTrials.gov and WHO ICTRP searches [68]. Among these, Moderna’s mRNA-4157/V940 (NCT03897881) and BioNTech’s BNT122 (autogene cevumeran) (NCT05142189) represent personalized neoantigen vaccines in melanoma, non-SCLC, and pancreatic carcinoma. The study outcome of phase 2 results demonstrated a 44% reduction in recurrence risk when combined with pembrolizumab compared to checkpoint blockade alone [116–119]. These vaccines deliver synthetic mRNA encoding TAAs to dendritic cells (DCs), which process and present the antigens on major histocompatibility complex (MHC) class I molecules, activating CD8+ T cells to target and destroy cancer cells. The vaccines offer several advantages, such as the ability to present multiple epitopes, bypass HLA type limitations, and enable rapid, scalable production without the need for nuclear localization. However, challenges remain, including the identification of tumor-specific neoantigens, optimizing delivery methods, and managing immune overactivation [120]. Recent work has also showcased a leading example of an LNP‑delivered, pan‑KRAS siRNA formulation encapsulated in microparticles, designed to target multiple *KRAS* mutations beyond *G12C*. In this context, the SIL‑204 siRNA exerts its antitumor activity in locally advanced pancreatic cancer [121, 122]. These data support the biological feasibility of *KRAS*-targeted siRNA strategies in pancreatic cancer and provide a translational framework for the future development of clinically viable *KRAS*-directed RNA therapeutics.

RNA-based CAR-T cell therapies offer significant benefits over traditional viral vector approaches, as they enable transient CAR expression and reduce the risk of severe adverse effects associated with permanent genetic modifications [123]. Electroporation is currently the most common method for delivering CAR-encoding mRNA into T cells, while innovative delivery systems such as LNPs, exosomes, polymer-based particles, and peptide transduction domains are actively being explored [124, 125]. Additionally, gene editing technologies like CRISPR/Cas9 and Cas13 offer precise, durable, and customizable treatments, presenting advantages over traditional therapies. Unlike siRNA-based therapies, which provide only transient suppression of gene expression, CRISPR directly edits DNA, ensuring permanent correction of targeted mutations [126]. The CRISPR-Cas9 system, along with newly discovered Cas12 and Cas13, holds significant potential for both gene editing and RNA-targeted therapies and diagnostics [127]. Concisely, further research and clinical trials are necessary to fully evaluate the safety, efficacy, and long-term outcomes of CRISPR-based therapies.

RNA-based therapies are currently in pre-clinical or early clinical development, and their potential to target the genetic drivers of challenging cancers positions them as a promising avenue for future FDA-approved treatments. Advances in safety, nanoparticle delivery, and next-generation chemistries are also expected to further enhance their therapeutic impact. Looking ahead, RNA therapeutics are anticipated to revolutionize personalized medicine by enabling customized treatments tailored to individual patients’ genetic mutations or resistance mechanisms, thereby improving outcomes and reshaping the landscape of recalcitrant cancer therapy. Finally, Table 4 presents an updated summary of all FDA-approved RNA therapeutics, their indication, molecular targets, and delivery modality.

**Table 4:** Food and Drug Administration (FDA) and/or European Medicines Agency (EMA) approved RNA drugs are currently in clinical use.

**RNA type**	**Drug**	**Brand name**	**FDA approval**	**Company**	**Indication**	**Delivery system**	**Target gene**
ASO	Fomivirsen	Vitravene	1998 (Withdrawn)	Ionis/Novartis	CMV retinitis	Naked	*IE2*
	Mipomersen	Kynamro	2013 (Withdrawn)	Kastle/Ionis	HoFH	Naked	*APOB-100*
	Nusinersen	Spinraza	2016	Ionis/Biogen	SMA	Naked	*SMN2*
	Eteplirsen	Exondys	2016	Sarepta	DMD	Naked	*DMD*
	Inotersen	Tegsedi	2018	Ionis	hATTR-PN	Naked	*TTR*
	Volanesorsen	Waylivra	2019	Ionis	FCS	Naked	*APOC3*
	Golodirsen	Vyondys 53	2019	Sarepta	DMD	Naked	*DMD*
	Viltolarsen	Viltepso	2020	Nippon Shinyaku	DMD	Naked	*DMD*
	Casimersen	Amondys 45	2021	Sarepta	DMD	Naked	*DMD*
	Tofersen	Qalsody	2023	Ionis/Biogen	SOD1-ALS	Naked	*SOD1*
	Eplontersen	Wainua	2023	AstraZeneca/Ionis	hATTR-PN	GalNAc	*TTR*
	Imetelstat	Rytelo	2024	Geron Corporation	MDS	Naked	*hTERT*
	Olezarsen	Tryngolza	2024	Ionis	FCS	GalNAc	*APOC3*
siRNA	Patisiran	Onpattro	2018	Alnylam	hATTR-PN	LNP	*TTR*
	Givosiran	Givlaari	2019	Alnylam	AHP	GalNAc	*ALAS1*
	Lumasiran	Oxlumo	2020	Alnylam	PH1	GalNAc	*HAO1*
	Inclisiran	Leqvio	2021	Novartis	HoFH	GalNAc	*PCSK9*
	Vutrisiran	Amvuttra	2022	Alnylam	hATTR-PN	GalNAc	*TTR*
	Nedosiran	Rivfloza	2023	Novo Nordisk	PH1	GalNAc	*LDHA*
Aptamer	Pegaptanib	Macugen	2004 (Withdrawn)	Pfizer/Eyetech	wAMD	PEG	*VEGF*
	Avacincaptad pegol	IZERVAY	2023	Archemix/Iveric Bio	GA	PEG	C5 protein

AHP: acute hepatic porphyria; ALAS1: aminolevulinic acid synthase 1; ASO: anti-sense oligo; CMV: cytomegalovirus; DMD: Duchenne muscular dystrophy; FCS: familial chylomicronemia syndrome; GA: geographic atrophy; GalNAc: *N*-acetylgalactosamine; HAO1: hydroxyacid oxidase 1; hATTR-PN: hereditary transthyretin-mediated amyloidosis with polyneuropathy; HoFH: homozygous familial hypercholesterolemia; LDHA: lactate dehydrogenase A; MDS: myelodysplastic syndromes; PH1: primary hyperoxaluria type 1; siRNA: short-interference RNA; SMA: spinal muscular atrophy; SOD1-ALS: amyotrophic lateral sclerosis caused by superoxide dismutase 1 mutation; VEGF: vascular endothelial growth factor; wAMD: wet age-related macular degeneration. Adapted with permission from Biopharma PEG: https://www.biochempeg.com/article/410.html#:~:text=Nucleic%20acid%20drugs%2C%20also%20known,are%20siRNA%20and%20ASO%20therapies. Accessed November 8, 2025. © Biopharma PEG Scientific Inc.

## Conclusions

Decades ago, RNA therapeutics development efforts were hindered by the perceived stability, commercially large-scale production, targeted delivery, and immunogenicity issues of RNA [111, 128, 129]. Recent scientific advances have resolved these issues; now, scientists have invented ways to stabilize RNA transcripts in the form of chemical modifications and/or circularization [130, 131]. Likewise, during the COVID-19 pandemic, techniques and machinery have been developed to manufacture GMP-grade RNA-based biologics in tens of millions of doses [132, 133]. Moreover, modified nucleotides (pseudouridine and N1-methylpseudouridine) currently used in RNA drug development have been discovered to have greatly reduced immunogenicity [134]. More importantly, nanotechnology has evolved to successfully deliver RNA drug candidates to targeted tissues in biological systems using methods such as aptamer and antibody-functionalised nanocarriers [135, 136]. Therefore, resources abound for oncology scientists, pharmaceutical experts, and clinical oncology professionals to exploit without reservation to stem the menace of recalcitrant and rare cancers. On another note, Brown et al. [137] reported that the average clinical development time for an FDA-approved innovative drug is 9.1 years. It has been 13 years since the “Recalcitrant Cancer Research Act” was passed into law [1]. Of note, Brown et al. [137] investigated a period between 2010 and 2020; out of 405 innovative drugs considered in their study within these 10 years, only 9 drugs were oligonucleotide-based (both RNA and DNA). More astonishing is the fact that 126 drugs were oncology drugs [137]. This example depicts the reality of treatment modalities receiving the majority of the monstrous research and subsequent clinical drug development effort. In this current review, we have attempted to present an insight into the potential of scientifically exploiting RNA drug development to complement outstanding ongoing research efforts towards combating recalcitrant and rare cancers.

More succinctly, the development of RNA therapeutics reflects decades of advances in RNA biology and molecular medicine, transforming RNA molecules into versatile therapeutic tools. Historical breakthroughs in RNA mechanisms have enabled the creation of diverse platforms, including ASOs, siRNAs, mRNA vaccines, and RNA-guided editing technologies, many of which have achieved clinical success in non-malignant diseases. Despite these achievements, translation into oncology, particularly for rare and recalcitrant cancers, remains constrained by limited molecular characterization, delivery challenges, safety concerns, and clinical trial limitations. Nevertheless, ongoing innovations in multi-omics profiling, RNA engineering, and targeted delivery systems are rapidly expanding therapeutic possibilities, positioning RNA-based strategies as promising future interventions for difficult-to-treat malignancies.

**Figure 3:**
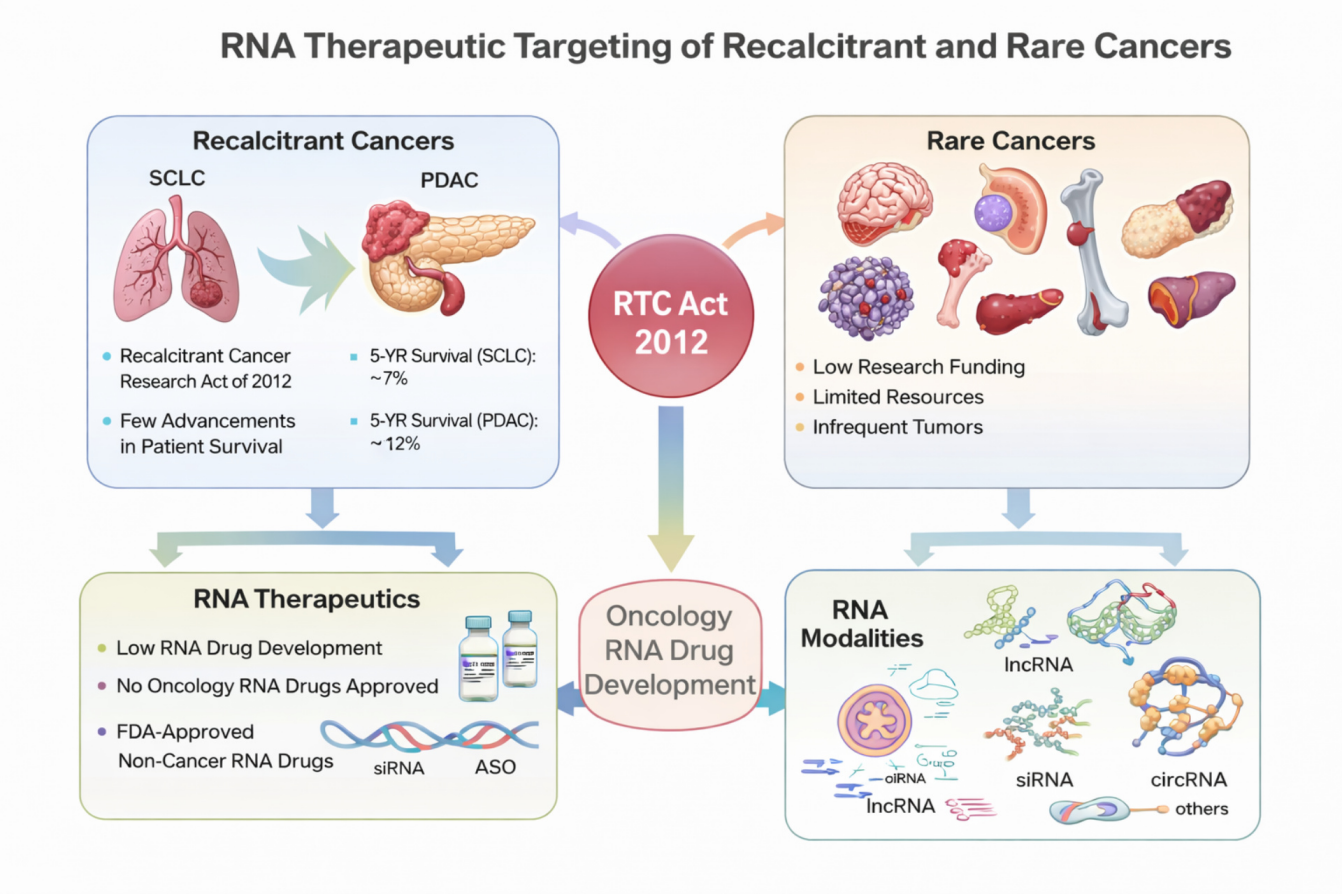
Summary figure showing the prospect of RNA drug development in recalcitrant and rare cancers.

In conclusion, tremendous research work has been done in investigating better therapeutic interventions for recalcitrant cancers since 2012. Consequently, leading to a couple of FDA-approved new and/or modified SCLC drugs. Clinically, a reduction is being observed in the incidence rate of SCLC in the USA, but the 5-year survival improvement recorded to date is limited. Of note, all old and new SCLC and other recalcitrant cancers are either small-molecule or protein drugs. The current review is drawing attention to the potential of exploring RNA drugs in combating the recalcitrant cancers menace. Hence, contributing to the potential improvement of recalcitrant cancer patients’ survival (Figure 3).

## Abbreviations

AHP: acute hepatic porphyria
ALAS1: aminolevulinic acid synthase 1
AMD: age-related macular degeneration
ASO: antisense oligonucleotide
CAR-T: chimeric antigen receptor T-cell therapy
circRNA: circular RNA
CRISPR-Cas: clustered regularly interspaced short palindromic repeats-associated protein
DMD: Duchenne muscular dystrophy
ES-SCLC: extensive stage small cell lung cancer
EURACAN: Expert Care for Rare Adult Solid Cancer
FDA: Food and Drug Administration
GalNAc: *N*-acetylgalactosamine
GBM: glioblastoma
hATTR: hereditary transthyretin-mediated amyloidosis
ICTRP: International Clinical Trials Registry Platform
*KRAS*: Kirsten rat sarcoma viral oncogene homolog
lncRNA: long non-coding RNA
LNP: lipid nanoparticle
LS-SCLC: limited-stage small-cell lung cancer
miRNAs: microRNAs
MYC: MYC family protein
NCI-MATCH: National Cancer Institute’s Molecular Analysis for Therapy Choice
NGS: next-generation sequencing
PD-1: programmed cell death protein 1
PDAC: pancreatic ductal adenocarcinoma
PD-L1: programmed death-ligand 1
PH1: primary hyperoxaluria type 1
RNAi: RNA interference
*SARS-CoV-2*: severe acute respiratory syndrome coronavirus 2
SCLC: small-cell lung cancer
siRNA: small interfering RNA
SMA: spinal muscular atrophy
*SMN1*: survival motor neuron 1
TAAs: tumor-associated antigens
TAPUR: Targeted Agent and Profiling Utilization Registry
VEGF: vascular endothelial growth factor
WHO: World Health Organization

## Declarations

### Copyright

© The Author(s) 2026.
